# Self-controllable prodromal symptoms of syncope attributed to carotid sinus syndrome during the end stage of cancer: a case report

**DOI:** 10.1186/s13030-016-0078-0

**Published:** 2016-09-05

**Authors:** Hideaki Hasuo, Kenji Kanbara, Hiroko Sakuma, Rie Matsumori, Mikihiko Fukunaga

**Affiliations:** Department of Psychosomatic Medicine, Kansai Medical University, Shinmachi 2-5-1, Hirakata, Osaka 573-1090 Japan

**Keywords:** Carotid sinus syndrome, Prodromal symptoms of syncope, End stage of cancer, Self-control, Coping

## Abstract

**Background:**

Carotid sinus syndrome (CSS) can cause prodromal symptoms of syncope such as dizziness and nausea. Patients with end-stage cancer lose self-efficacy associated with reduced activities of daily life (ADL). Herein, we report a case of end-stage cancer in which self-efficacy was enhanced as the patient gained self-control of prodromal symptoms of syncope.

**Case presentation:**

A 70-year-old patient with end-stage esophageal cancer and enlarged supraclavicular lymph nodes developed CSS. The CSS was a mixed type with both bradycardia and decreased blood pressure, accompanied by prodromal symptoms prior to syncope episodes. The patient incidentally discovered that he could decrease the duration of symptoms by contracting the muscles in his hands and legs. By applying this coping method at the onset of prodromal symptoms, he was also able to reduce the severity and duration of symptoms, which resulted in enhanced self-efficacy. As a result, the frequency of prodromal symptoms also decreased even though ADL improved.

**Conclusion:**

This patient was diagnosed with vasoinhibitory-predominant mixed-type CSS. The coping method the patient developed seemed to avoid the onset of abrupt blood pressure decrease via peripheral vascular constriction action. Achievement of adequate coping such as self-control of prodromal symptoms enabled our patient to improve his self-efficacy even at the end stages of cancer. This case of enhanced self-efficacy could possibly illustrate a placebo effect for prevention of recurrence.

## Background

Cases of advanced cervical tumor are sometimes associated with syncope. One of the causes of syncope is secondary carotid sinus syndrome (CSS), which is attributed to the stimulation of carotid sinus baroreceptors by the tumor [1, 2]. Cases of CSS are usually diagnosed when patients have cerebral ischemic episodes secondary to carotid sinus hypersensitivity. Prodromal symptoms of syncope, such as dizziness and nausea, followed by a syncope episode are characteristic features of CSS. Based on circulatory disorder patterns, CSS is classified into three different types: cardio-inhibitory type, in which a carotid sinus massage produces asystole exceeding 3 s; vasodepressor type, in which systolic blood pressure decreases by 50 mmHg or more; and the mixed type where both disorders are observed [3]. Neck rotation, extension or pressure can be triggers for cerebral ischemic episodes, all which can affect the patient’s autonomy [3]. The radical cure for neck tumor-associated carotid sinus hypersensitivity includes surgical tumor resection and tumor reduction by chemo- or radiotherapies [4]; however, their application is difficult in cases of end-stage cancer. It was reported that pacemaker implantation may be effective for the treatment of the cardio-inhibitory type CSS [5]. No standard therapy has been established for the vasodepressor type, and there have been few reports about efficacy of medications [6, 7]. Additionally, careful consideration about potential invasive treatment or medication is required for end-stage cancer patients. The behavioral changes patients make to cope with certain problems and symptoms is called “coping.” Adequate coping, such as self-control, has recently received a lot of attention as a psychological intervention for stress reduction. In early specific palliative care, supporting patients’ coping methods has been addressed as a critical intervention [8]. Further, it has been reported that patients’ self-control and management of symptoms can improve the self-efficacy of cancer patients [9, 10].

Our search found no reports about patient self-control for prodromal symptoms of syncope caused by secondary CSS. Furthermore, our case illustrates a reduced frequency of prodromal symptoms concomitantly with improved ADL and self-efficacy. Herein, we describe a patient who was able to decrease the severity, duration, and frequency of prodromal symptoms of syncope associated with secondary CSS by adequate coping.

## Case presentation

### Patient background

The patient was a 70-year-old male with a history of glaucoma. There was no outstanding family medical history. He was diagnosed with esophageal cancer 3 years earlier, and had undergone surgical resection and chemotherapy. Best supportive care was initiated 1 year earlier. From 1 month before the last hospitalization, the patient had been in the terminal stage of cancer with an Eastern Cooperative Oncology Group performance status of 3 and decreased activities. At that point, he was told he had 1–2 months to live. Around the same time, the left supraclavicular lymph node was becoming progressively enlarged. Consequently, the patient developed repetitive syncope episodes lasting 2–3 min for which hospitalization was necessary.

**Fig. 1:**
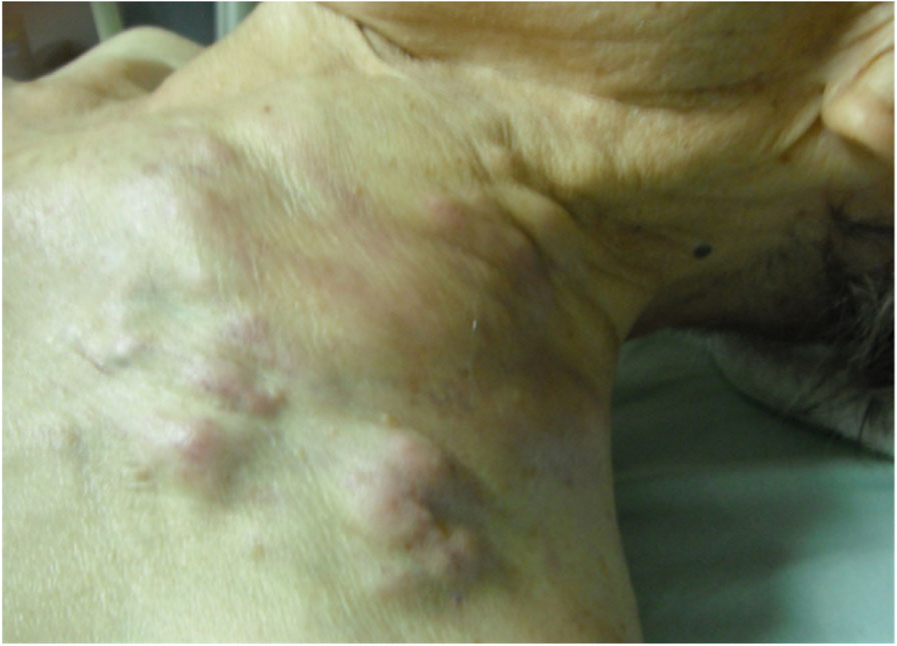
A single tumor mass in the supraclavicular area

A single mass of 120 mm × 90 mm was detected in the supraclavicular area on palpation (Fig. 1). No outstanding findings were detected with cardiac ultrasonography or cerebral magnetic resonance imaging. No blood biochemistry findings suggestive of anemia, dehydration, hypoglycemia, electrolyte abnormalities or thyroid disorder were observed.

### Diagnosis of carotid sinus syndrome

**Fig. 2:**
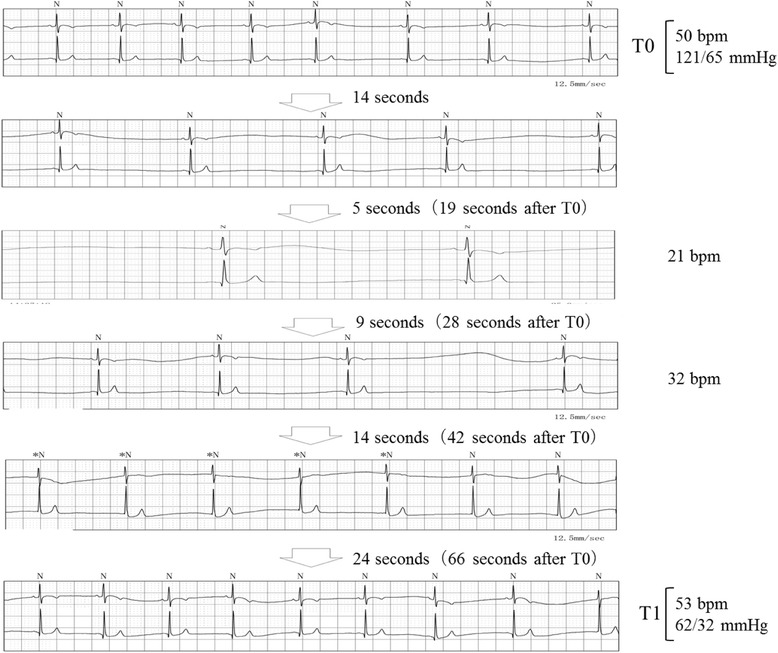
Holter electrocardiography during prodromal symptoms of syncope

After admission, ADL decreased and the patient became bedridden because of his anticipatory anxiety for syncope onset. Subsequently, syncope episodes disappeared; however, the patients complained of prodromal symptoms such as dizziness, nausea, and ocular pain. These symptoms persisted for 20–30 min, spontaneously disappeared, and recurred three to four -times daily. When prodromal symptoms occurred, the heart rate ranged between 20 and 30 bpm and systolic blood pressure decreased by 50 mmHg. Holter electrocardiography performed on the day after admission revealed stable blood pressure and heart rate as long as no syncope occurred. During a syncope episode, however, abrupt bradycardia persisted for 1–2 min, initially with a non-sinus rate, then returning to a sinus rate (Fig. 2). This cycle recurred five to six -times in 20–30 min. During this period, arrest for more than 2 s was frequently observed, and the recovery time in the sinus node was within 5 s. One of the factors contributing to the prodromal symptoms was cervical rotation toward the tumor side. Although the head-up tilt test could not be performed owing to the patient’s poor health condition, postural change with angles between 0° and 80° were performed using a motorized bed. The postural changes did not induce prodromal symptoms or changes in blood pressure or heart rate, which did not support a diagnosis of vasovagal syncope. Although the tilt table test with carotid sinus massage was avoided because of the patient’s poor health condition, the patient was diagnosed with a mixed-type secondary CSS based on the syncope episodes.

### Patient’s decision-making for medical intervention

The patient understood his status well, including the poor prognosis. On admission, he expressed that he knew he might live a few weeks at best. His will was not to undergo life-prolonging treatment but to obtain symptom relief and respect for his autonomy. In particular, he wished for reduction of the severity of the prodromal symptoms and improvement of his autonomy by reducing anticipatory anxiety. He declined to receive palliative radiation therapy to the cervical tumor or implantation of a temporary or permanent pacemaker because of their invasive nature and his poor prognosis.

### Coping with prodromal symptoms of syncope

Palliative radiation therapy to the cervical tumor was initially considered; however, it was not warranted based on the patient’s will and because the effects of this treatment could take some time to be reflected in the patient’s condition and because this treatment was associated with poor prognosis. Implantation of a temporary or permanent pacemaker was also initially considered; however, it was not warranted based on the patient’s will as well as the suspected vasoinhibitory-predominant mixed-type [11]. Although treatment with α_1_ adrenergic agonist was initially considered, it was not warranted based on the patient’s history of swallowing disorder due to recurrent laryngeal nerve paralysis associated with the cervical tumor.

On the fourth day of hospitalization, the patient discovered that he could decrease the duration of prodromal symptoms by contracting the muscles in his hands and legs, by clenching his hands into fists and continuously stomping his feet on his bed while lying in it. In particular, symptoms that had previously persisted for 20–30 min were resolved in several tens of seconds by applying his coping method. When he tried to cope with the prodromal symptoms, his heart rate decreased up to 20–30 bpm; however, no change in blood pressure was observed. He hoped for improvement of his autonomy and discovered this coping method incidentally and with the support of medical staff. Based on his experience, he recognized these actions as effective coping and applied them whenever he experienced prodromal symptoms.

### Clinical course after initiation of coping (Fig. 3)

**Fig. 3:**
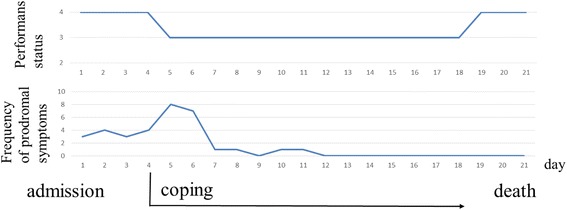
Clinical course of prodromal symptoms of syncope after initiation of coping

Because coping reduced the severity and duration of prodromal symptoms, the patient recognized amelioration of the prodromal symptoms. Subsequently, his anticipatory anxiety for syncope was reduced, resulting in enhanced self-efficacy. He had demonstrated improved ADL, including being able to elevate his head with the motorized bed, maintaining a sitting position and performing cervical rotation as early as 1 day after the initiation of coping. As ADL improved, the frequency of prodromal symptoms transiently increased up to seven or eight times a day during the early phase of coping. The frequency thereafter decreased and eventually the prodromal symptoms subsided after 4 days of coping. Nineteen days after admission, he developed sudden aspiration pneumonia secondary to recurrent laryngeal nerve paralysis associated with the cervical tumor and decreased awareness. He had not complained of prodromal symptoms and had maintained his ADL status for the 19 days after admission. On day 21 after admission, he died from respiratory failure.

### Discussion

As far as we know, this is the first report of a case of end-stage cancer that led to enhanced self-efficacy with self-control of prodromal symptoms of syncope associated with secondary CSS. There are two critical points in this case. First, prodromal symptoms of syncope associated with CSS were successfully self-controlled. Second, the frequency of prodromal symptoms of syncope decreased after self-control became effective.

The first critical point in this case was that prodromal symptoms of syncope associated with CSS were successfully self-controlled. The hypothesis that the tumor could induce depolarization in afferent and efferent nerve fibers was a possible underlying mechanism of CSS in this case of cervical tumor; however, details regarding this hypothesis have not yet been elucidated. Efferent nerve fibers involved in the carotid sinus reflex split into the cardiac vagal nerve, which is distributed to the sinus and the atrioventricular nodes. The sympathetic nerve is distributed to the ventricular myocardium and peripheral blood vessels [12]. Depending on the abnormality of the regulating function, CSS is classified as cardio-inhibitory type when the stimulation of the vagal nerve inhibits the sinus node function or atrioventricular conduction, or a vasodepressor type when the inhibition of the synthetic nerve reduces blood pressure [3]. In our case, we feel that the patient’s coping method, consisting of contracting the muscles in his hands and legs, induced is peripheral vasoconstriction followed by enhanced synthetic nerve function, which avoided an abrupt decrease in blood pressure. Indeed, based on the Holter electrocardiogram, we observed that an abrupt decrease in blood pressure by 50 mmHg or greater occurred within 66 s after the bradycardia emerged. In contrast, after his coping method was applied, there were no decreases in blood pressure although comparable bradycardia occurred. Based on these episodes, this patient was considered to present a vasoinhibitory-predominant mixed-type CSS. As this patient was not monitored with direct measurement of arterial pressure, no continuous blood pressure data were available. It was previously reported that blood pressure showed the lowest value 18 ± 3 s after the carotid sinus massage in a vasoinhibitory-type CSS [3]. Similarly, blood pressure might have decreased rapidly in our patient after cervical rotation. Coping in our case was considered to have either avoided the abrupt decrease in blood pressure or contributed to rapid recovery from hypotension. Additionally, it was reported that interruption of cerebral circulation for 6–8 s or a decrease in systolic blood pressure to 60 mmHg can result in syncope [13]. Thus, the recovery period in the sinus node might be within 5 s, thus, bradycardia alone was not considered to be a primary factor for the prodromal symptoms of syncope.

The second critical point from this case was that not only the severity of prodromal symptoms of syncope decreased but also the frequency of prodromal symptoms decreased gradually after self-control became effective. Although the frequency of prodromal symptoms transiently increased with improved ADL in the early phase, the symptoms had almost resolved 4 days after the initiation of coping. This amelioration was considered to be due to decreased subjective symptoms associated with increased threshold for the prodromal symptoms of syncope. It was reported that the preventive effects of pacemaker treatment against recurrence of syncope can be attributed to a placebo effect caused by pacemaker implantation [14]. Namely, self-efficacy enhanced by self-control might contribute to a placebo effect that increased the threshold of symptoms. As for pain management, it was reported that a patient’s recognition of self-efficacy that can reduce pain enhances analgesic potency [9]. Additionally, in an experimental study of end-stage cancer patients, the existence of stress associated with decreased ADL was observed [15]. Coping in this case might have led to stress reduction caused by increased ADL, possibly resulting in an increased threshold of symptoms. Furthermore, tilt training was reported to prevent the recurrence of neurally mediated syncope [16]. Coping in this case led to an increased duration of sitting due to improved ADL, which might possibly work as a preventive exercise against the recurrence of prodromal symptoms of syncope.

## Conclusions

Prodromal symptoms of syncope in CSS were successfully self-controlled. This patient was considered to present a vasoinhibitory-predominant mixed-type CSS. The coping was considered to have avoided abrupt decrease in blood pressure via peripheral vascular constriction action. Both severity and frequency of prodromal symptoms of syncope decreased after self-control became effective. In this case, our patient achieved adequate coping, namely self-control that led to enhanced self-efficacy, even in the end-stage of cancer. This enhanced self-efficacy could possibly reflect a recurrence prevention effect associated with a placebo effect. In cases of prodromal symptoms of syncope associated with vasoinhibitory-predominant CSS, self-control such as the coping method used by our patient may be effective.

## Abbreviations

ADL: Activities of daily life
CSS: Carotid sinus syndrome
ECOG: Eastern Cooperative Oncology Group
MRI: Magnetic resonance imaging
